# Acknowledgement to Reviewers of *Journal of Functional Biomaterials* in 2017

**DOI:** 10.3390/jfb9010005

**Published:** 2018-01-12

**Authors:** Journal of Functional Biomaterials Editorial Office

**Affiliations:** MDPI AG, St. Alban-Anlage 66, 4052 Basel, Switzerland

Peer review is an essential part in the publication process, ensuring that *Journal of Functional Biomaterials* maintains high quality standards for its published papers. In 2017, a total of 52 papers were published in the journal. Thanks to the cooperation of our reviewers, the median time to first decision was 18 days and the median time to publication was 43 days. The editors would like to express their sincere gratitude to the following reviewers for their time and dedication in 2017:
Akhavan, BehnamKramer, PhillipAl-Dujaili, EmadKundu, JoydipAlem, HalimaLebaron, RichardAmorati, RiccardoLee, Hae-HyoungAniello, FrancescoLee, WonhyeArnaud, FrançoiseLee, WooCheolAtrens, AndrejLlena, CarmenBiglino, GiovanniŁukomksa-Szymańska, MonikaBouropoulos, NikolaosMadeddu, RobertoBrantley, William A.Maeda, HirotakaBrauer, Jeremy A.Mahapatro, AnilBusquets, Maria AntòniaMandava, NandaCalvo-Guirado, José LuisMao, ChuanbinCapasso, RaffaeleMarpu, Sreekar BabuCervadoro, AntonioMartin, Thomas JohnChang, Thomas M.S.Martins, AlbinoChang, Wen-WeiMaruyama, KeiChou, JoshuaMathew, MathewCicciu, MarcoMayer, ChristianCollins, Maurice N.McCoy, Sarah J. BreeseColtelli, Maria BeatriceMekonnen, Tizazu H.Costa-Rodrigues, JoãoMelo, Mary Anne S.Cristea, CeciliaMenabue, LediDe Aza, PiedadMendes, Ana C.Do, Sun HeeMizusaki, MasanobuDoloff, Joshua C.Naito, YoshihitoEggenhöffner, RobertoNoubissi, FeliciteEsposito, CiroOh, Daniel S.Farsad, KhashayarOkamoto, MasamiFauzi, IlianaOskouei, RezaFernandes, Susana C.M.Palumbo, Fabio SalvatoreFeyerabend, FrankPapini, AlessioFigueiredo, MarxaPartonen, TimoFinley, Brent L.Patterson, JenniferForner, LeopoldoPenarrocha-Diago, MiguelGentile, PiergiorgioPerepelyuk, MarynaGirousi, StellaPetridis, LambisGizdavic-Nikolaidis, Marija R.Piluso, SusannaGlaser, RainerPistone, AlessandroGolkowski, MarkPucci, AndreaGómez-Florit, ManuelQuencer, KeithGracia, IgnacioRadecki, JerzyGrandfield, KathrynRanzato, EliaGranet, RobertRavaioli, StefanoGraziani, GabrielaRen, YanfangGrenho, LilianaRohnke, MarcusHaapasalo, MarkusRoldo, MartaHadjiargyrou, MichaelRossouw, Paul E.Hayakawa, TohruSantos, JoaoHerndon, DavidSebastian, AimyHildreth, BlakeSeeliger, ClaudineHirner, Alfred V.Sgarbossa, PaoloHu, JackSharma, BheshHu, YangShavandi, AminIntaglietta, MarcosShie, Ming-YouIrie, MasaoShih, Yu-RuJennings, JessicaSilikas, NikolaosKevadiya, BhaveahSilva, Simone S.Khandaker, MorshedSimoni, JanKim, Chun HoSiracusa, ValentinaKim, Hyoung SeopSpriano, SilviaKim, Kyung-suStamboulis, ArtemisKim, Tae ImStan, GeorgeKim, Young-JinSurmenev, Roman A.Knipping, KarenSwaminathan, ViswanathanKoizumi, HiroyasuTakeshita, HaruoKootala, SujitTaubenberger, AnnaKoźlecki, TomaszTavri, Sidhartha

